# Opinion: Medical education in many low- and middle-income countries needs urgent attention and serious improvement

**DOI:** 10.3389/fmed.2025.1548112

**Published:** 2025-03-26

**Authors:** Sandro Vento

**Affiliations:** ^1^Faculty of Medicine, University of Puthisastra, Phnom Penh, Cambodia; ^2^Department of Clinical Disciplines, Manash Kozybayev North Kazakhstan University, Petropavlovsk, Kazakhstan

**Keywords:** medical education, low and middle income countries (LMICs), medical schools, apprenticeship, competency based medical education

## Introduction

Medical education has been extensively debated and repeatedly modified in Western countries since the 1910 Flexner Report recommended a transformation of U.S.A. medical schools. It is debatable whether the changes have ultimately been beneficial to those countries; however, a pressing issue is whether medical education is as effective as it should be in low- and middle-income countries (LMICs), where most doctors are “produced.” It is mandatory that medical education is at a sufficient level in all medical schools of all countries; is that the case at the present time?

In this Opinion article, I will briefly examine the main aspects which need serious attention and urgent improvement.

## Is the medical schools' mission fulfilled?

Are medical schools in practice (on paper, they may be) a lively and creative environment for teaching, innovation, and patient care? Most are not; they do not have the funds, staff and overall conditions to do so. Their so-called tripartite mission (research, teaching, patient care) is untenable in most low- and middle-income countries, as physicians cannot do well in all three. Most of the universities are unable to start any research project unless they obtain large grants, and faculty members involved in these efforts have no or very little time to spend with students. In addition, clinical faculty mainly focus on training residents rather than undergraduates.

The inalienable, paramount mission of a medical school must be to form competent and compassionate doctors. Herbert Fred provided 20 years ago an excellent description of the increasingly overlooked importance of history taking and clinical skills for any clinician, and advocated for American students' clinical experience to take place largely in real-world settings, supervised by experienced, compassionate, common-sense practitioners (1). This continues to be extremely important for medical students in LMICs.

## How to attract doctors to an academic career?

For a medical school to fulfill its mission, it is fundamental to attract the brightest and most compassionate doctors. How to succeed in this? Doctors should work in adequately staffed primary care clinics or university hospitals, have adequate time for bedside and small group teaching, and earn salaries comparable to those of physicians who devote most of their time to private practice. Medical schools in LMICs cannot continue to rely largely on part-time or even casual doctors, as this unacceptably lowers the quality and consistency of student teaching.

## Which should the focus of the undergraduate curriculum be?

In September 1978, in Almaty (then Alma Ata), the World Health Organization (WHO) defined primary health care as essential (2); management of complex chronic diseases, early diagnosis and disease prevention were considered of paramount importance. However, too much emphasis has since been put on tertiary, highly specialized care; disease prevention, primary care and the fundamental value of patient-doctor relationships are, *de facto*, overlooked, even though much emphasized theoretically. It is essential that they are given the importance that they still have, and must also have in the future, for medicine to continue to contribute to societal wellbeing. Therefore, the undergraduate medical curriculum must truly focus on primary care rather than on highly specialized tertiary hospital care.

## Apprenticeship vs. simulation-based education

Simulation-based training has been hugely promoted in medical education in the current millennium; notwithstanding its much-emphasized benefits, the high costs, need for specialized equipment and facilities, and faculty training remain important barriers (3). It is essential that simulation equipment ensures realism of the experience by reflecting the often limited resources in the clinical environment in LMICs. In any case, clinical experiences on real patients must also be guaranteed, and increased; the traditional apprenticeship model (students learning through direct patient care under proper supervision of experienced clinicians) should be strongly pursued rather than increasingly disregarded. Professor Greenhalgh stated almost 10 years ago that “we need to challenge the colonization of medical education by the unreal: the simulated patient, the silicone body part, the standardized scenario, the objective and structured (but entirely fictitious) clinical examinations,” and that “the inexorable retreat of medical education from the messy, non-standardisable reality of illness and suffering” should be stopped (4). Her words should be seriously considered even nowadays.

## Is medicine a “calling” or a business?

Self-interest, family and/or friend pressure, and socioeconomic status influence the students' choice of medicine worldwide (5–7). In the past, medicine was chosen because young people wished to help, earn a high salary and be respected by others; presently, not only a high salary but also flexible working hours are essential for millennial students (8), and the idea of working long hours for inadequate public sector salaries is unacceptable. In Indonesia, for instance, medical students prefer jobs with a positive work-life balance; in addition, they would like to have jobs where more technology and more procedural protocols are used, teamwork is involved, and cases are not too complicated (9). Primary care is not an interesting career option for most of them (9). It is mandatory to consider the above reality to find ways to increase interest in primary care during undergraduate studies, and it is also compulsory to enormously improve working conditions in the public health sector in LMICs (10).

## Student selection and teaching methodologies

A career in medicine is not for all. Medical schools should utilize a combination of tools to select their students, including academic performance measures (where the fact that scores at fee-paying private schools are higher than those at state or public schools should be considered), cognitive skills tests, semi-structured or multiple mini-interviews. Assessment of personal qualities must be an essential component of the admission process (11), as failures in professionalism are often linked to earlier problematic behaviors by students (12). Still practiced, direct entrance of high school graduates into medical schools, based exclusively on their performances in few basic subjects (13), must be abandoned in all countries.

Due consideration must be given to the knowledge of English, which has been for decades the international language of medicine (14) and a fundamental international educational tool; weakness in English has long been recognized as a hindrance to medical students' communication and information handling skills (15). Considering the difficulty of medical studies, it is important to seriously consider the inclusion of English proficiency among the pre-requisites for admission rather than eventually providing English lessons during undergraduate medical studies. The emphasized need to favor access to medical schools of students from disadvantaged communities/background must not cause relaxation of necessary tools; what is mandatory is to truly favor their access to good primary and secondary schools.

West-inspired theories such as student-centered approach and problem-based learning (PBL) are theoretically attractive but cannot be easily applied and could even be detrimental in other settings. For instance, if there are insufficient numbers of faculty members and facilitators and/or a faculty is made exclusively or almost exclusively of casual lecturers, PBL cannot be applied or, if tentatively applied, will be a failure. Importantly, in most countries in Asia, Africa, Latin America and the Mediterranean, where collectivistic societies strongly avoid uncertainty, and hierarchical cultural backgrounds prevail, lecturers are expected to make learning decisions for their students (16). Even in Japan, the implementation of PBL is problematic, due especially to the high burden placed on faculties, and is therefore avoided in many medical schools (17). As for assessment, in Asia neither students nor lecturers are ready for more dialogical and formative assessment (16). In fact, students place importance on getting certified achievements in (largely multiple-choice, question-based) summative assessments, rather than on self-directed activities or peer-review (17). Hence, studies comparing outcomes of different teaching methodologies and assessment types are needed in LMICs. It is also very important not to try to implement competency-based medical education in medical schools of LMICs without very seriously and carefully considering its numerous challenges [faculty development, increased faculty workload, increased faculty time, need for improved educational infrastructure and technology support, need for comprehensive training programs and continuing support for students and faculty members (18)].

## Cheating in summative assessments

Academic integrity must be absolutely safeguarded in medical school exams. Is this the case? Unfortunately, it is not. Dishonesty is quite common among undergraduate medical students during online assessments (19, 20), and cheating is frequent also in theses (21). This behavior, if known to the general public, would be considered totally unacceptable; in contrast, students and lecturers have low expectations of each other's behavior (22). Medical schools should be much more seriously held accountable to produce competent doctors for the countries where they are located and for the world at large.

## Minimum requirements of medical schools

Since 1998, the World Federation for Medical Education (WFME) has developed global standards for quality improvement, which have been accepted and tentatively applied in many countries. However, nearly 80% of medical colleges in India (the country with the largest number of medical colleges in the world) do not meet even the minimum criteria established by the regulator National Medical Commission; faculty absenteeism, inadequate and poor infrastructure, poor student-teacher relationship are major issues (23). In addition, increasing commercialization and privatization of medical education, unabated corruption, relaxation of basic requirements for establishing new medical schools, flawed entrance selection processes are huge challenges (24). The situation is not dissimilar in other countries. Does accreditation solve all problems and guarantee that competent doctors come out of every medical school? It does not, but the lack of it tends to guarantee the opposite.

## Hospital and clinic placement of medical students

This is an extremely important issue. The students' clinical training must happen in conducive environments where there is proper attention to/care of the students. All medical schools must ensure this, and it is essential that clinicians in primary care clinics and hospitals are linked with medical schools through agreements that guarantee due attention to teaching and student supervision. Each medical school must have a budget and pay clinicians for their contribution. Ministries of health must ensure that the staffing of hospitals and clinics where medical students rotate is sufficient to make adequate clinical training possible, and that the salaries paid for their clinical activity are such that the clinicians do not spend most of their time in private practice, even when officially not allowed. All the above requires a shift of mindset that is anyway necessary if medical education is considered in real terms as important as it must be.

## Need for practicing clinicians and career choices

LMICs' investment in medical education should not be in vain. A study involving 959 fifth- and sixth-year students in six government owned medical schools in Ethiopia in 2015 indicated that only 70.1% wanted to practice in clinical care settings (25). This situation is not uncommon; clinicians who have even specialized in different fields in Western countries end up doing only administrative work in the ministries of health, hospitals or medical schools in LMICs, and their clinical expertise is lost. As for those who choose to work clinically, it is important that each ministry of health carefully plans the number of doctors needed in primary care and in the various specialties, and that strong cooperation is in place with all medical schools in the country. The lack of a sufficient number of doctors in primary care [even more so in rural areas (25)] and in specialties which are considered less remunerative is an unsolved issue that needs strong attention and innovative approaches.

## Conclusions

I have briefly examined the huge problems and deficiencies of medical education in many LMICs; some of them are common also to Western countries, even though largely ignored. Obviously, not all medical schools in all LMICs are affected by the outlined problems and deficiencies, and some schools are actually preparing their students for medical practice very well. However, it is imperative to try and hugely improve all medical schools, and it is up to governments to ensure that clinicians educated in any medical school of their nations are competent, knowledgeable, compassionate and able to take care of the whole population. It is also important for everybody to consider that healthcare must not be a business but rather a fundamental right for every human being in any country; medical schools should prepare clinicians who will keep this in mind.

## References

[1] Fred HL. Hyposkillia: deficiency of clinical skills. Tex Heart Inst J. (2005) 32:255–7. 16392201 PMC1336689

[2] World Health Organisation. Declaration of Alma-Ata (Paragraph VI, page 1). International Conference on Primary Health Care, Alma-Ata, USSR, 6–12 September 1978. World Health Organisation, Geneva (1978). Available online at: https://www.who.int/publications/i/item/WHO-EURO-1978-3938-43697-61471 (accessed December 19, 2024).

[3] Elendu C Amaechi DC Okatta AU. The impact of simulation-based training in medical education: a review. Medicine. (2024) 103:e38813. 10.1097/MD.0000000000038813 38968472 PMC11224887

[4] Rimmer A. Medical schools overuse simulation technology, says leading academic. BMJ. (2015) 350:h2427. 10.1136/bmj.h2427

[5] Faizullina K Kausova G Grjibovski A. Every third Kazakhstani medical student regrets the choice of education: a cross-sectional survey in Almaty. Ethiop J Health Dev. (2013) 27:235–42.

[6] Jothula KY Ganapa P Sreeharshika D Naidu NK Abhishek P. Study to find out reasons for opting medical profession and regret after joining MBBS course among first year students of a medical college in Telangana. Int J Community Med Public Health. (2018) 5:1392–6. 10.18203/2394-6040.ijcmph20180983

[7] Ibrahim Bashir MM Fadelalla Alrayah MA Elsayed Mustafa ME Abdulla Maroof MK Omer Hamad MA Ali Mohamedosman MM. Medicine as a career choice: a comprehensive study on factors influencing Sudanese students to opt in/out medical career. BMC Med Educ. (2023) 23:418. 10.1186/s12909-023-04415-w 37287048 PMC10246522

[8] Gillissen A Kochanek T Zupanic M Ehlers JP. Millennials medical students generation at the crosswalks: motivations and attitudes towards study and future career - a mixed-method study. Adv Med Educ Pract. (2022) 13:1305–19. 10.2147/AMEP.S368128 36281458 PMC9587722

[9] Hikma HN Mora MC Ova OE. Students' motivation in choosing general practice for their career pathway: a middle-income country report, Indonesia. Int J Med Educ. (2022) 13:56–63. 10.5116/ijme.6209.1974 35247875 PMC9017499

[10] Vento S. Medicine should be learned in a conducive environment. Lancet. (2023) 401:267–8. 10.1016/S0140-6736(23)00009-0 36709070

[11] Powis D. Selecting medical students: an unresolved challenge *Med Teach*. (2015) 37:252–60. 10.3109/0142159X.2014.993600 25532428

[12] Papadakis MA Teherani A Banach MA Knettler TR Rattner SL Stern DT. Disciplinary action by medical boards and prior behavior in medical school. N Engl J Med. (2005) 353:2673–82. 10.1056/NEJMsa052596 16371633

[13] Lim S Cheab S Goldman LN Ith P Bounchan Y. The past, present and future of medical education in Cambodia. Med Teach. (2024) 46:842–8. 10.1080/0142159X.2024.2327490 38493077

[14] Maher J. English as an international language of medicine. Med Educ. (1987) 21:283–4. 10.1111/j.1365-2923.1987.tb00363.x 3626893

[15] McLean M Murdoch-Eaton D Shaban S. Poor English language proficiency hinders generic skills development: a qualitative study of the perspectives of first-year medical students. J Furth High Educ. (2013) 37:462–81. 10.1080/0309877X.2011.645461

[16] Lee CY Jenq CC Chandratilake M Chen J Chen MM Nishigori H. A scoping review of clinical reasoning research with Asian healthcare professionals. Adv in Health Sci Educ. (2021) 26:1555–79. 10.1007/s10459-021-10060-z 34254202 PMC8610955

[17] Shimizu I Nakazawa H Sato Y Wolfhagen IHAP Könings KD. Does blended problem-based learning make Asian medical students active learners?: a prospective comparative study. BMC Med Educ. (2019) 19:147. 10.1186/s12909-019-1575-1 31092243 PMC6521359

[18] Bhattacharya S. Competency-based medical education: an overview. Ann Med Sci Res. (2023) 2:132–8. 10.4103/amsr.amsr_27_2333692645

[19] Dar UF Khan YS. Self-reported academic misconduct among medical students: perception and prevalence. ScientificWorldJournal. (2021) 2021:5580797. 10.1155/2021/5580797 34475809 PMC8407971

[20] Pongchaikul P Vivithanaporn P Somboon N Tantasiri J Suwanlikit T Sukkul A. Remote online-exam dishonesty in medical school during COVID-19 pandemic: a real-world case with a multi-method approach. J Acad Ethics. (2024). 10.1007/s10805-024-09571-2

[21] Khadem-Rezaiyan M Dadgarmoghaddam M. Research misconduct: a report from a developing country. Iran J Public Health. (2017) 46:1374–8. 29308381 PMC5750349

[22] Tonkin AL. “Lifting the carpet” on cheating in medical school exams. BMJ. (2015) 351:h4014. 10.1136/bmj.h4014 26286467

[23] Sharma P. Four out of five medical colleges fail medical education regulator's minimum standards. Mint (2024). Available online at: https://www.livemint.com/news/india/four-out-of-five-medical-colleges-fail-medical-education-regulators-minimum-standards-11717911484869.html (accessed December 19, 2024).

[24] Gupta VK Gupta M Gupta V. Challenges in medical education. Curr Med Res Pract. (2022) 12:73–7. 10.4103/cmrp.cmrp_16_22

[25] Assefa T Haile Mariam D Mekonnen W Derbew M. Medical students' career choices, preference for placement, and attitudes towards the role of medical instruction in Ethiopia. BMC Med Educ. (2017) 17:96. 10.1186/s12909-017-0934-z 28558753 PMC5450253

